# Non-functioning paraganglioma of the urinary bladder: A case report and review of the literature

**DOI:** 10.3892/ol.2014.1790

**Published:** 2014-01-10

**Authors:** YONGQING LAI, DUQUN CHEN, ZUHU YU, LIANGCHAO NI, SHANGQI YANG

**Affiliations:** 1 Department of Urology, Peking University Shenzhen Hospital, Shenzhen PKU-HKUST Medical Center, Shenzhen, Guangdong 518036, P.R. China; 2 Guangdong and Shenzhen Key Laboratory of Male Reproductive Medicine and Genetics, Institute of Urology, Peking University Shenzhen Hospital, Shenzhen PKU-HKUST Medical Center, Shenzhen, Guangdong 518036, P.R. China; 3 Anhui Medical University, Hefei, Anhui 230032, P.R. China

**Keywords:** paraganglioma, urinary bladder, therapeutics, prognosis

## Abstract

Paragangliomas are extra-adrenal tumors of the autonomic nervous system and may be found within the skull base, neck, mediastinum and periaortic region. Paragangliomas of the urinary bladder are rare, and non-functioning bladder paraganglioma is even rarer and not easily recognized. Histological examination is often key in leading to a definitive diagnosis. The current report presents a case of a 28-year-old female with urinary bladder paraganglioma. The patient presented with no classical signs and symptoms, and these were only appreciated following histological examination of a transurethral resection specimen that elucidated the correct diagnosis. In the present report, the clinical features, diagnosis, management and pathological observations of paraganglioma of the urinary bladder are discussed.

## Introduction

Paraganglioma is a neoplasm that develops from the chromaffin tissue of the sympathetic nervous system situated outside the adrenal medulla (1). It is also referred to as extra-adrenal pheochromocytoma. Paragangliomas of the urinary bladder account for <1% of all bladder tumors and 6% of all extra-adrenal pheochromocytomas (2). Bladder paraganglioma is similar to adrenal pheochromocytoma, the majority of which secrete catecholamines and cause symptoms of pheochromocytoma and bladder tumors, including headache, palpitations and fainting, which are particularly associated with micturition and hematuria. Paraganglioma of the urinary bladder is rarely encountered and its biological behavior is uncertain. In addition, the prognosis of bladder paraganglioma has not been well established. The present study describes a 28-year-old female with non-functioning paraganglioma of the urinary bladder and presents a supplementary review of the previously published cases and literature.

## Case report

**Figure 1:**
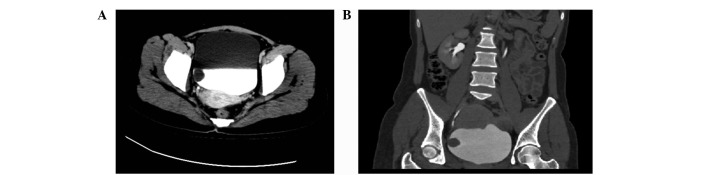
Axial and coronal views from computed tomography scan revealing the position of the tumor protruding into the bladder with an intact surface.

A neoplasm of the urinary bladder was identified during a routine examination of a 28-year-old female patient, three days later the patient was admitted to the Peking University Shenzhen Hospital (Shenzhen, China). The patient had no abdominal symptoms, had been in good health, with no previous medical problems, and had no specific family past medical or drug history. The patient did not complain of headache and had no history of hypertension. Routine hematological examination and biochemical tests were within normal limits and physical examination showed no evidence of hypertensive disease. Ultrasonography revealed an abnormal mass on the right lateral wall of the urinary bladder and computed tomography (CT) of the abdomen showed a solitary tumor protruding into the bladder. This was confirmed by cystoscopy demonstrating a protruding tumor on the right wall of the bladder with normal mucosa, measuring 2.0×2.0 cm (Fig. 1A and B). No sign of any metastatic disease was found on ultrasound examination or CT scans of other abdominal organ systems. On the basis of the first diagnosis of bladder tumor, a transurethral resection was performed and the procedure was uneventful with no hypertension or occurrence of massive bleeding. Postoperative recovery was good and at three months follow-up the patient felt well, with no signs of recurrence.

### Pathological examination of the tumor indicated paraganglioma

**Figure 2:**
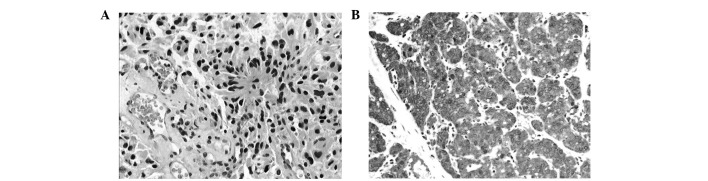
Histopathological evaluation of the paraganglioma. (A) Paraganglioma composed of dual cell populations arranged in a characteristic nested Zellballen pattern (H&E staining; magnification, ×400). (B) Synaptophysin (immunohistochemical stain) confirmed neuroendocrine origin, compatible with paraganglioma (magnification, ×400).

On histopathological evaluation, the tumor cells were arranged in a nested pattern (Fig. 2A). The histopathological examination showed positive staining for NSE, Syn, CgA and CD56, sustentacular cells stained positive for S-100 and the Ki-67 staining revealed a proliferation index of <2%, compatible with paraganglioma (Fig. 2B). Written informed consent was obtained from the patient. The collection and use of clinical data were reviewed and approved by the Peking University Shenzhen Hospital (Shenzhen, China) Ethics Committees.

## Discussion

Pheochromocytoma is a rare tumor with an estimated 800 cases diagnosed yearly in the USA, of which ~10% are extra-adrenal or paragangliomas (1). The most common sites for paraganglioma include the carotid body, jugular foramen, mediastinum, organ of Zuckerkandl and periaortic region (3). Paraganglioma of the urinary bladder is rarely encountered and non-functioning bladder paraganglioma is even rarer. The first case of bladder paraganglioma was reported in 1953 (4). The underlying mechanism of bladder paraganglioma remains unclear. Previous studies have shown that bladder paraganglioma occurs more frequently in females than males (female/male ratio is 3:1), primarily during the second and third decades of life (5). The majority (83%) of paragangliomas of the urinary bladder are hormonally active (6). The main symptoms include headache, tachycardia, sweating and paroxysmal hypertension, particularly during micturition. Therefore, when the presence of paraganglioma of the urinary bladder is suspected, endocrine tests must be performed, including catecholamines and vanillylmandelic acid in a 24-h urine sample, serum epinephrine and others. However, non-functioning paragangliomas are rarer and more difficult to diagnose due to their non-secreting nature (6). Clinically, the patient provided no history of hypertension, headache or flushing that would suggest a diagnosis of paraganglioma. Therefore, endocrine screening for paraganglioma was not considered in the management of the patient. However, not all patients have typical test results if blood or urine samples are not collected at the occurrence of typical symptoms.

Preoperative location and qualitative analysis are extremely important in confirming the diagnosis. B ultrasound, CT and magnetic resonance imaging may be of great use in localizing the tumor (7–9). Ultrasound shows the tumor as a submucosal homogeneous mass, with a clear outline, continuous mucosa, abundant blood supply and a possible cystic or necrotic appearance at its center. CT shows the association between the mass and bladder mucosa and muscular and peri-tissue (7,8). I131-methyliodobenzylguanidine (MIBG) is an analog of norepinephrine and is absorbed by the paraganglioma tissue. I131-MIBG has been used in diagnosing and localizing extra-adrenal paraganglioma with a specificity close to 100% and a sensitivity approaching 90% (10). Positron emission tomography (PET) offers even higher accuracy than MIBG scans in the localization of paragangliomas due to the higher spatial resolution of PET scanning (11,12). The tumors in cystoscopy appear as globular submucosal masses protruding into the bladder with an intact surface, continuous mucosa and abundant blood supply. However, the significance of cystoscopy is limited as, when the tumor is functional, it greatly induces blood pressure fluctuations and causes difficulties when the bladder is irrigated, unless the necessary medicines and instruments are available. Biopsy under cystoscopy is not recommended since it has a low positive rate, risk of bleeding and may provoke a hypertensive crisis.

For the majority of asymptomatic bladder paragangliomas, a definitive diagnosis may be reached only by histology. The tumors show histological features similar to adrenal pheochromocytomas and the cells usually grow in a characteristic nested Zellballen pattern. Immunohistochemical staining is required for a definitive diagnosis. Chromogranin, synaptophysin and NSE may aid the identification of neural tissue and neuroendocrine cells (13). A positive staining with synaptophysin, NSE, CD56, NSE, Syn, CgA and S-100 was observed in the present case, which was compatible with paraganglioma. Between 5 and 15% of the paragangliomas of the urinary bladder are said to be cancerous. However, no histological criteria have been established to distinguish between benign and malignant tumors. Only the appearance of local invasion or distant metastases confirms that the tumor is cancerous (14).

The majority of paragangliomas are sporadic in nature, but ~10% of these tumors may be associated with genetic disorders, such as familial paraganglioma, neurofibromatosis type 1, von Hippel-Lindau, Carney triad and multiple endocrine neoplasia type 2 (15). Therefore, it has been suggested that all patients with extra-adrenal or multifocal pheochromocytoma, or a family history, must undergo genetic testing.

The most effective management is surgical resection, including transurethral resection and partial or total cystectomy. The perioperative preparation and treatment may not be simplified, particularly for patients who exhibit characteristic paroxysmal hypertension during micturition. It is necessary to stabilize hypertension prior to surgery, using α-blocking agents (phenoxybenzamine) or calcium channel blockers for two weeks to inhibit the release of catecholamine and expand the blood volume, which is similar to the management for adrenal pheochromocytoma. Occasionally, it is difficult to determine a definitive preoperative diagnosis, resulting in insufficient preparation, which complicates the transurethral resection since unexpected intraoperative hypertensive crisis and bleeding may occur (16). With the advances in laparoscopy techniques, laparoscopic partial cystectomy has become the treatment of choice. However, the optimal management mode remains uncertain. Due to the multilayer involvement of the bladder wall, open surgery to perform partial cystectomy is recommended. Transurethral resection is considered to be feasible in tumors of <2 to 3 cm in size without deep parietal infiltration (2). In the present case, a transurethral resection was successfully performed. Intraoperative blood pressure remained stable during surgery and the margins were negative for tumor. Radiation therapy has been advocated for patients who are unable to undergo surgery or for unresectable tumors (17).

Regular follow-up is necessary to detect late recurrences (18). It must be life-long and include cystoscopy, plasma or urinary tests and imaging study. No consensus has been established for the frequency of these measures; however, we suggested at least an annual follow-up for those patients who are asymptomatic or whenever clinically indicated.

Non-functioning bladder paraganglioma is easily misdiagnosed. Transurethral resection for bladder paraganglioma may be a treatment of choice, offering several advantages, including reduced invasion, rapid recovery and early discharge from the hospital, but the optimal management mode remains uncertain. A definitive diagnosis may be reached only by histology and no histological criteria have been established to distinguish between benign and cancerous tumors. Long-term annual follow-up is recommended in all paragangliomas.
